# Cavitary Hodgkin Lymphoma of the Lung

**DOI:** 10.7759/cureus.58152

**Published:** 2024-04-12

**Authors:** Emeka P Agudile, Ibrahim Elkhawas, Zeeshan Solangi, Frank Schembri, Kamran Manzoor

**Affiliations:** 1 Internal Medicine, Steward Carney Hospital, Boston, USA; 2 Social and Behavioral Sciences, Harvard T.H. Chan School of Public Health, Boston, USA; 3 Pulmonary and Critical Care Medicine, Baystate Medical Center, Springfield, USA; 4 Pulmonary and Critical Care Medicine, South Shore Health, Weymouth, USA

**Keywords:** classical hodgkin, nodular subtype hodgkin lymphoma, excisional biopsy, pulmonary lymphoma, cavitary lung lesions

## Abstract

It has been shown that some cases of Hodgkin lymphoma (HL) may present with pulmonary parenchymal involvement usually in the form of multiple irregularly marginated pulmonary nodules. Other radiographic patterns such as consolidation, interstitial infiltrates, and cavitary lesions are less common. We present a case of HL, nodular sclerosis type, with pulmonary involvement presenting as a large cavitary consolidation and axillary and mediastinal lymphadenopathy. Initial diagnostic work-up including sputum culture, bronchoscopy, and a fine needle aspiration of lymph node was not conclusive favoring a reactive process with a presumptive diagnosis of cavitary pneumonia. A follow-up chest imaging revealed worsening right upper lung mass, axillary adenopathy, and new nodular satellite lesions, and a repeat bronchoscopy with multiple biopsies remained non-diagnostic requiring an excisional biopsy of the axillary lymph node confirming HL. Further transthoracic core biopsies of the cavitary lung lesion were consistent with pulmonary lymphoma involvement.

## Introduction

Both Hodgkin lymphoma (HL) and non-Hodgkin lymphoma (NHL) may present with pulmonary manifestations. The precise incidence and prevalence of pulmonary involvement by lymphoma is not known. About 15-40% of HL and about 4% of NHL are reported to present with pulmonary parenchymal involvement by some studies [1,2]. Other studies reported a prevalence of 38% of HL and 24% of NHL. Characteristically, HL present in the lungs as multiple masses or nodules ranging from 2 mm to 100 mm on imaging, but presentations in the forms of consolidation or cavitary lesions are relatively rare [3,4].

We present a case of HL, nodular sclerosis type, with pulmonary involvement presenting as a large cavitary consolidation and detail the difficulties in establishing the diagnosis. 

This article was previously presented as a meeting poster at the CHEST 2023 Annual Scientific Meeting on October 10, 2023.

## Case presentation

**Figure 1:**
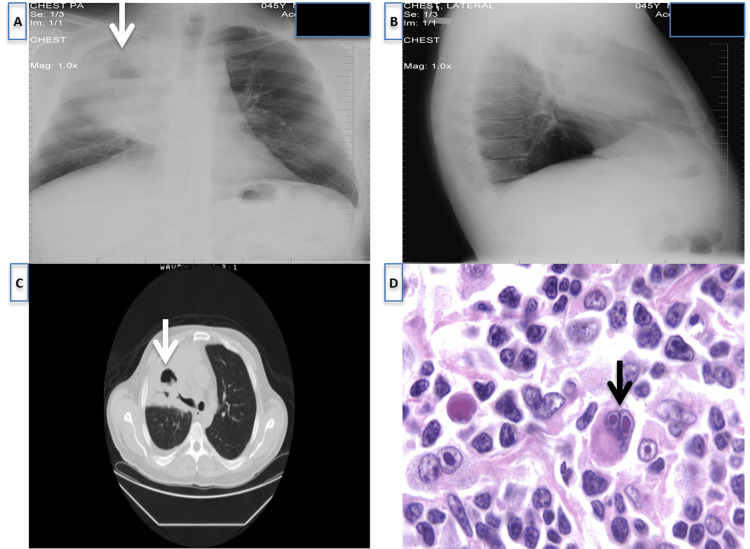
Chest X-rays (A) AP view and (B) lateral view showing large RUL thick-walled cavitary lesion with AFL (white arrow). (C) Chest CT shows a large infiltrate of almost the entire RUL with AFL (white arrow). (D) Histopathology micrograph showing proliferation of small lymphocytes, neutrophils, eosinophils, plasma cells, and large atypical cells characteristic of Reed-Sternberg cells (black arrow). RUL: right upper lobe; AFL: air-fluid level

A 45-year-old man with a history of positive purified protein derivative (PPD) presented to the emergency room with several weeks of cough, night sweats, and a 40-pound weight loss. He appeared cachectic, with poor dentition and a 2.5 cm right axillary lymph node. He had normal labs other than a leukocytosis of 20.1 with predominant neutrophilia. Imaging with chest X-ray (CXR) and chest computed tomography (CT) scan showed a large right upper lung cavitary infiltrate, with right axillary and mediastinal lymphadenopathy (Figure 1). Sputum acid-fast bacillus (AFB) was negative, and culture yielded *Moraxella catarrhalis*. A bronchoscopy showed compression of the right upper lobe (RUL) with cytology showing atypical squamous cells. A fine needle aspiration (FNA) biopsy of the axillary lymph node favored a reactive process. A presumptive diagnosis of cavitary pneumonia was made, and the patient was discharged on a prolonged course of antibiotics.

**Figure 2:**
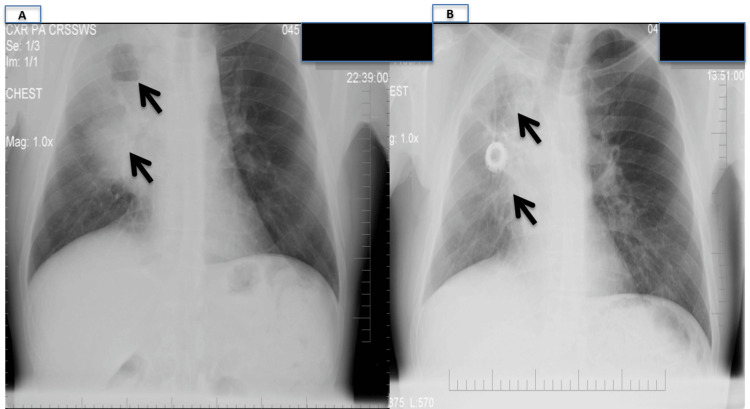
Chest X-rays (A) before and (B) after treatment was started and completed (black arrows).

He returned two months later with worsening dyspnea. A repeat chest CT showed an increase in the RUL mass, enlarging axillary adenopathy, and new nodular satellite lesions. A repeat bronchoscopy showed progressive compression of the right-sided segments, but multiple biopsies and brushings were non-diagnostic. Ultimately, an excisional biopsy of the axillary lymph node showed large Reed-Sternberg cells confirming HL. Further transthoracic core biopsies of the cavitary lung lesions showed that 20% of cellularity are lymphocytes (88% are T cells, CD4/CD8=4.1, B3 (polyclonal, K/L=1.5, NK=8)). Immuno-histologic evaluation revealed large, atypical cells CD15+, CD20+, CD30+ (weakly), CD3-, and LCA equivocal. Background lymphocytes were CD3+. This was consistent with pulmonary parenchymal involvement by HL, and a diagnosis of classic Hodgkin lymphoma (CHL), nodular sclerosis type, was made. The patient was initiated on the doxorubicin (Adriamycin), bleomycin, vinblastine, and dacarbazine (ABVD) regimen with significant radiographic improvement (Figure 2).

## Discussion

CHL commonly involves the mediastinal lymph nodes, and pulmonary parenchymal involvement is less common [1,5,6]. Pulmonary involvement has been noted to occur in about 15-40% of HL cases [1,5,6]. It has been reported that pulmonary infiltrates are observed in 20% of cases at the outset of the disease, 40% during the clinical course, and 60% at autopsy [4]. Pulmonary infiltrate is almost always associated with hilar or mediastinal lymphadenopathy in HL [6], and the pathogenic basis is thought to be due to lymphatic spread from regional lymph nodes or through hematogenous dissemination [4].

Broad categories of patterns of lung involvement have been described including large masses, mass-like consolidation, and nodules with or without cavitation, ground-glass, and lymphangitis/perilymphatic patterns [1,7]. The most common pattern is a mass or mass-like consolidation of the lung greater than 1 cm with or without cavitation [2]. Cannonball metastases, multiple small nodules resembling metastatic disease, and multiloculated cavitary lesions are sometimes encountered but are rare occurrences in CHL [2,6]. Bilateral pulmonary infiltrate due to CHL has also been described [7,8]. Solitary or multiple cavitary lung lesions due to HL are very rare and occur in less than 1% of cases [9,10].

This case highlights a rare presentation of pulmonary HL, with a large cavitary consolidation. The differential diagnosis of such cavitary presentation is complex and may include but is not limited to conditions such as lung abscess, or necrotizing pneumonia, metastatic carcinoma, eosinophilic granulomatosis with polyangiitis, tuberculosis, septic emboli, fungal infections, and sarcoidosis [9,11]. This mimicry of a wide range of disease conditions makes the diagnosis of a cavitary lung lesion secondary to HL challenging and prone to delayed diagnosis or misdiagnosis as lung abscess, necrotizing pneumonia, or pulmonary tuberculosis.

Notable in this case were the multiple non-diagnostic biopsies, with the diagnosis ultimately being made via an excisional lymph node biopsy. This is consistent with prior literature suggesting improved yield for lymphoma via core needle or surgical excisional biopsies. As a result, HL or other lymphomatous involvement of the lung is an important differential in either cavitary or non-cavitary infiltrates, especially those that persist or worsen despite anti-infective therapy and elude attempts at diagnostic biopsies [9,10]. On the other hand, alternative etiologies such as tuberculosis, aspergillosis, granulomatosis with polyangiitis, sarcoidosis, and rheumatic nodules should be considered if there are multiple or multiloculated cavitary lung lesions [6]. Cavitary lesions in HL have been postulated to be due to central ischemic necrosis, presumably secondary to rapid tumor growth especially in large nodules and masses [3]. A cavity with air-fluid levels results when there is communication between an adjacent bronchus and a necrotic tumor mass. Also, it has been observed that cavitary lesions due to HL appear to be resistant to radiotherapy with poorer prognosis [3].

## Conclusions

Pulmonary parenchymal involvement in HL usually presents with pulmonary nodules but can include more unusual radiographic presentations such as a cavitary infiltrate. This diagnosis can be difficult to make on standard bronchoscopy. HL should, therefore, be considered an important differential diagnosis of cavitary pulmonary lesions, and core needle or surgical excisional lymph node biopsies should be considered.
